# Association of Lower COMT Activity Alleles with Aggressive Traits in Male Youth with Conduct Disorder Living in a Correctional Facility

**DOI:** 10.3390/biom15040554

**Published:** 2025-04-09

**Authors:** Lucija Tudor, Josip Podobnik, Gordana Nedic Erjavec, Matea Nikolac Perkovic, Jaanus Harro, Margus Kanarik, Darko Marcinko, Dubravka Svob Strac, Melita Cusek, Vlatka Kovac, Nela Pivac

**Affiliations:** 1 Rudjer Boskovic Institute, Division of Molecular Medicine, 10000 Zagreb, Croatia; lucija.tudor@irb.hr (L.T.); gnedic@irb.hr (G.N.E.); mnikolac@irb.hr (M.N.P.); dsvob@irb.hr (D.S.S.); 2 Department of Psychiatry, Psychiatric Hospital for Children and Youth Zagreb, 10000 Zagreb, Croatia; josippodobnik@yahoo.com; 3 Juvenile Correctional Facility Ivanec, 42240 Ivanec, Croatia; melita.cusek@mrosp.hr; 4 Neuropsychopharmacology, Institute of Chemistry, University of Tartu, 50411 Tartu, Estonia; jaanus.harro@ut.ee (J.H.); margus.kanarik@ut.ee (M.K.); 5 Department of Psychiatry, Clinical Hospital Center Zagreb, 10000 Zagreb, Croatia; niarveda@gmail.com; 6 Department for Child and Adolescent Psychiatry, Clinical Hospital Center Osijek, 31000 Osijek, Croatia; vlatka.kovac@yahoo.com; 7 University of Applied Sciences Hrvatsko Zagorje Krapina, 49000 Krapina, Croatia

**Keywords:** aggression, association, conduct disorder, correctional facility, *COMT* rs4680 and rs4818 polymorphisms, genetic variants, male youth

## Abstract

Aggression constitutes a significant behavioral issue associated with delinquent behavior, commonly observed in youth diagnosed with conduct disorder (CD) and living in correctional facilities. Catechol-o-methyl-transferase *(COMT)* gene variants modify the environmental sensitivity associated with the risk of aggression. This study evaluated the association of *COMT* rs4680 and rs4818 polymorphisms with aggressive behavior in 341 male adolescents living inside and outside a correctional facility, with or without a diagnosis of CD. Aggression was assessed using the Hare Psychopathy Checklist: Youth Version (PCL-YV), Modified Overt Aggression Scale (MOAS) and Swanson, Nolan and Pelham Questionnaire. *COMT* rs4680 and rs4818 polymorphisms were genotyped using TaqMan SNP Genotyping Assays. A similar prevalence of the *COMT* genotypes or haplotypes was found between adolescents with or without CD, suicidal behavior, or detention in correctional facility. In youth with CD, the *COMT* rs4680 A allele was associated with higher MOAS verbal aggression, aggression toward objects, irritability (subjective and open), and PCL-YV interpersonal domain scores compared with carriers of the *COMT* rs4680 G allele. *COMT* rs4818 GC carriers with CD had higher scores on the MOAS subjective irritability than GG heterozygotes. These novel findings revealed the association of lower *COMT* rs4680 and rs4818 activity alleles with aggression in detained male adolescents with CD.

## 1. Introduction

In youth with psychopathic traits and psychopathy-related behaviors, sex differences in conduct disorder (CD) and clustering of CD symptoms have been reported [1]. CD affects 8% of adolescents, with a prevalence in males (11%) compared to females (7%) [2]. CD is assumed to be related to psychopathy in adulthood, including adult antisocial behavior [3], delinquency, and adult criminal behavior [4]. In half of the affected youth, CD is associated with the callous–unemotional (CU) trait [5,6], with distinct symptoms, such as reduced guilt, callousness, limited prosocial emotions, lack of remorse or empathy or concern about school performance, shallow or deficient affect, and uncaring behavior. In addition, youth with CD frequently develop antisocial and disruptive behavior that can manifest as violence, aggressive and non-aggressive rule-breaking, abuse of societal norms, and disrespect for the rights and properties of others. These behaviors are more frequently found in male youth [7]. Consequently, the signs of CD can be detected in adolescence in the form of destructive and violent behaviors, lying, stealing, and cruelty to animals and people, all of which might lead to delinquency, criminality, and the development of antisocial personality disorder [8]. Additionally, CD is frequently comorbid with substance abuse or attention deficit/hyperactivity disorder (ADHD), and individuals with CD comorbid with ADHD or early-onset substance use exhibited more severe aggressive behaviors, psychopathic traits, violent recidivism, history of violent crimes, and had worse outcomes than individuals without CD, ADHD, or substance use [9]. A meta-regression analysis and systemic review enrolling adolescents in juvenile detention and correctional facilities (N = 32,787) observed that in 61.7% of males and 59.0% of females with a CD diagnosis, there was a significant association with delinquency and aggressive behavior [10]. This confirmed that the prevalence of CD is significantly higher in detained adolescents than in the general population of adolescents in the US [2,10].

Environmental risk factors for CD include a lack of parental support, the presence of parent and child problems, and disrupted relationships [11]; living in a juvenile correctional facility [10,12,13] or in juvenile detention [14] might also present a risk factor for CD and aggression, as well as for the lifetime prevalence of psychiatric morbidity [15,16,17]. Aggression is usually subdivided into proactive (or predatory, focused on harming others) or reactive (as a reaction to a perceived threat) aggression [18]. Moreover, aggressive behavior is often related to a “fast” life strategy, adopted to counteract harsh, hostile, and unpredictable home environments that contribute to more severe maladaptive behaviors, such as increased substance use, criminal behavior, early pregnancy, and mental health problems [19]. These aggressive and rule-breaking behaviors are associated with criminal recidivism, and they are predominantly detected in youth living in correctional facilities, especially in those diagnosed with CD [9,10,20].

The neurobiological underpinning of aggressive behavior remains unclear, but the expression of psychopathic traits is assumed to be influenced by various biological systems, including catecholamines [21] and various associated genes [18,22]. Catechol-o-methyl transferase (*COMT*) metabolizes catecholamines and modulates dopamine function and is responsible for the majority of dopamine degradation in the prefrontal cortex (PFC), which is the brain region involved in dopamine-dependent cognitive task performance [23]. The *COMT* gene coding for the COMT enzyme is located on chromosome 22q11.2 [24]. The most frequently evaluated *COMT* polymorphism is a functional single-nucleotide polymorphism (SNP), Val158Met (rs4680), a substitution of guanine (G) with adenosine (A), resulting in Valine to Methionine change [25,26]. *COMT* rs4680 polymorphism results in three *COMT* genotypes: Val/Val (A/A), Val/Met (A/G), and Met/Met (G/G) [25], and it significantly affects COMT function and activity by impacting its thermal stability. Specifically, the *COMT* AA genotype is associated with reduced abundance, stability, and activity of the COMT enzyme [26], leading to slower dopaminergic degradation and, consequently, longer dopamine availability in the PFC synapses in carriers of the AA genotype when compared to GG homozygotes.

*COMT* rs4818 polymorphism is another commonly studied SNP that causes C/G substitution and results in the GG, CG, and CC genotypes. Although it is a synonymous polymorphism, with both alleles resulting in Leucine (Leu) residue [27], it is suggested that *COMT* rs4818 polymorphism contributes to the changes in COMT activity even more than *COMT* rs4680 polymorphism by altering the mRNA secondary structure that could affect the translation of the COMT protein [28]. The *COMT* rs4818 G allele has been associated with higher levels of S-COMT in PFC [29], higher COMT activity, lower PFC dopamine signaling, and less efficient planning and problem-solving ability when there is no emotional feedback, but it could be more favorable in decision-making, which includes emotional processing [27], and also with treatment resistance in schizophrenia [30] and severe negative symptoms and anhedonia in schizophrenia [31]. These associations are presumably due to its influence on the genes [32] involved in synaptic plasticity and cortical functioning through estrogen-mediated mechanisms, as COMT also metabolizes catechol-estrogens.

The *COMT* SNPs rs4818 and rs4680 are part of the haploblock (rs6269-rs4633-rs4818-rs4680) containing haplotypes that affect COMT enzymatic activity and pain sensitivity [33]. Research data suggest that predictions of COMT activity may be effectively narrowed to the rs4818-rs4680 micro-haplotypes, with the GG haplotype associated with higher COMT activity, and that the combined assessment of *COMT* rs4680 and *COMT* rs4818 haplotypes provides a high level of informativeness [34,35].

*COMT* rs4680 polymorphism has often been associated with different phenotypes and psychopathy-related behaviors in mental disorders, related to alterations in catecholaminergic [21] and especially dopaminergic [36] signaling. Therefore, the association of *COMT* rs4680 SNP with aggression and delinquency was frequently evaluated [4,37,38], while the other *COMT* rs4818 SNP was less frequently studied, but it has been reported to be related to particular aggressive traits in young children [39]. However, recent meta-analysis [4] revealed that the results linking aggression and *COMT* rs4680 are mixed and conflicting due to various confounding factors, such as environmental factors, sex, age, race, and different diagnostic assessments.

To exclude some of these confounding factors on the association between *COMT* rs4680 or rs4818 polymorphisms and aggression, the present study included only Caucasian male adolescents between 16 and 18 years of age, subdivided according to the diagnosis of CD, and according to living in or out of the correctional facility. Aggressive behavior was assessed using clinical and psychometric evaluations [40,41,42,43,44,45]. *COMT* rs4680 and rs4818 genotypes and haplotypes were controlled for the possible effects of sex, age, and smoking. We expected that *COMT* genetic variants would be associated with a diagnosis of CD, aggressive behavior, and/or living in a correctional facility. Our hypothesis was that the presence of the *COMT* low activity rs4680 A or rs4818 C allele, compared to the G allele, will be associated with aggression in youth who developed CD and who were living in a correctional facility.

## 2. Materials and Methods

### 2.1. Participants

This study enrolled 341 drug-free male adolescents from 16 to 18 years of age, of whom 120 had a diagnosis of CD and 221 were without CD, according to the Structured Clinical Interview for DSM-IV criteria [40], since a validated DSM-5 was not available in the Croatian language at the time. The inclusion criteria were male adolescents who volunteered to participate in this study with the consent of their guardians. All subjects with CD were detained in the Juvenile Correctional Facility Ivanec, Zagreb County, Croatia, while 65 control adolescents were recruited from the same correctional facility, as described earlier [41,42], although they did not meet the diagnostic criteria for CD (Table 1). The assessment of antisocial and aggressive behavior was performed using the Hare Psychopathy Checklist: Youth Version (PCL-YV) [43] and Modified Overt Aggression Scale (MOAS) [44,45] in both groups. The delinquency adjudication cases were characterized by a higher level of activities with the characteristics of a criminal offense, conduct disturbance syndrome with a deep-rooted antisocial lifestyle (the problem of antisocial behavior by DSM-IV criteria), a lower level of prior contact with psychiatric services, and less frequent use of psychopharmacotherapy in anamnesis. All subjects from the correctional facility underwent double clinical and psychometric evaluations by psychiatrists and a psychologist.

Additional age-matched, non-delinquent, and non-aggressive control young male subjects (N = 156) who were not living in detention, and who were recruited from the Estonian Children Personality, Behavior and Health Study organized by the University of Tartu, Estonia, were evaluated as non-aggressive (i.e., with 0 scores) based on the Swanson, Nolan and Pelham Questionnaire IV (SNAP-IV) scores and DSM-IV criteria [40]. These control young subjects were sampled in the schools of Tartu County, Estonia, as part of the Estonian Children Personality, Behavior and Health Study [46], representing a representative sample of the youth population [47]. Some of these subjects were enrolled in our previous study, as previously described [48]. The exclusion criteria were as follows: currently taking any medications or using psychoactive substances, treatment history of cognitive behavioral therapy or electroconvulsive therapy, diagnoses of autism spectrum disorder or ADHD, substance use disorder, other psychiatric diagnoses, intellectual disability, or a refusal to give consent to participate in the study voluntarily. This study was approved by the Ethics Committees of each participating institution and adhered to the ethical criteria outlined in the 1975 Helsinki Declaration.

### 2.2. Blood Processing

Blood samples were drawn in the morning using BD VacutainerTM glass collection tubes with the acid citrate dextrose (ACD) anticoagulant (Becton, Dickinson and Company, Franklin Lakes, NJ, USA) and processed on the same day. DNA from peripheral blood was isolated using a salting-out method [49] or a DNA isolation kit for mammalian blood (Boehringer Manheim, Biberach, Germany) and stored at −20°C until further analysis.

### 2.3. Genotyping of COMT Polymorphisms

Genotyping of the *COMT* Val158Met (rs4680) and rs4818 polymorphisms was performed by real-time PCR, using the ABI Prism 7300 Real-time PCR System apparatus (Applied Biosystems, Foster City, CA, USA), with primers and probes from Applied Biosystems (Foster City, CA, USA) as TaqMan^®^ SNP Genotyping Assays (SNP ID: C__25746809_50 (rs4680), C___2538750_10 (rs4818)) and according to the procedures described by Applied Biosystems. Briefly, initial denaturation (95 °C, 10 min) of approximately 30 ng DNA in 10 μL reaction volume was followed by 50 cycles of denaturation (92 °C, 15 s) and elongation (60 °C, 90 s). Aside from the codominant model, which included all three genotypes (AA, GA, GG for *COMT* rs4680 and GG, CG, CC for *COMT* rs4818), we have evaluated dominant models for *COMT* rs4680 (AA + GA vs. GG) and for *COMT* rs4818 (GG + CG vs. CC) polymorphisms. Genotyping was performed by the researchers, who were blinded to all clinical data. For quality control, 5% of all samples (blind duplicates) were genotyped again.

### 2.4. Statistical Analyses

Statistical analyses were performed using Prism version 7.00 (GraphPad Software, Inc., San Diego, CA, USA). Since the Kolmogorov–Smirnov test showed a significant deviation of data from the normal distribution, non-parametric tests (Mann–Whitney U test for two groups and Kruskal–Wallis ANOVA by ranks, followed by Dunn’s post hoc test, for the evaluation of three or more groups) were used to analyze differences in the severity of various parameters of aggression and antisocial behavior between carriers of different *COMT* genotypes and alleles. The results were reported as median and interquartile range, while significant results were represented by box-plot diagrams. Haploview 4.2 software [50] was used to determine LD values between the *COMT* rs4818 and rs4680 polymorphisms based on the confidence interval method [51]. Since the two *COMT* polymorphisms were in the strong linkage disequilibrium, or LD (D’ > 0.80), an expectation–maximization algorithm integrated into the PLINK 1.07 software [52] was used to assign the most probable haplotype pair for each individual. The χ^2^-test was used to analyze the differences between genotype, allele, and haplotype frequencies of *COMT* Val158Met and *COMT* rs4818 polymorphisms between groups. All tests were two-tailed, and since the two polymorphisms were evaluated, the *p*-value was adjusted to <0.025. G*Power 3 Software indicated that the study had an adequate sample size (N = 341) and appropriate statistical power to detect significant differences between the studied groups: for the Kruskal–Wallis ANOVA (with α = 0.025; power (1-β) = 0.800; medium effect size (ω = 0.25) and 4 groups), the total desired sample size was 180; for the genetic analyses and χ^2^-test (with α = 0.025; power (1-β) = 0.800 and medium effect size (ω = 0.30)), with df = 1, the total desired sample size was 106, and with df = 2, the total desired sample size was 128; and for the Mann–Whitney test (with α = 0.025; power (1-β) = 0.800; medium effect size (ω = 0.50)), the total desired sample size was 128.

## 3. Results

### 3.1. Demographic Data

The study included 341 male adolescents 16 to 18 years old (120 with a diagnosis of CD and 221 without CD). All 120 subjects with CD, and 65 control subjects without CD, were detained in the juvenile correctional facility (Table 1). The assessment of aggressive behavior was performed using the Hare Psychopathy Checklist: Youth Version (PCL-YV) and Modified Overt Aggression Scale (MOAS) in both groups, where the subjects with CD had significantly higher PCL-YV scores (U = 769.0; *p* < 0.001) and MOAS total scores (U = 1383.0; *p* < 0.001) than the control subjects and had more delinquency adjudications, although this finding was not significant (χ^2^ = 2.951; *p* = 0.086). Smoking was more prevalent among subjects with CD than in the control group (χ2 = 11.710; *p* < 0.001). Additional age-matched, non-aggressive control subjects (N = 156), with 0 scores on the Swanson, Nolan and Pelham Questionnaire IV (SNAP-IV), were recruited from schools in Tartu County and were not detained in the correctional facility (Table 1). Thus, the control group included both Croatian and Estonian subjects.

**Table 1:** Demographic and psychometric data of control subjects and subjects with CD.

	Control Subjects (N = 221)	Subjects with CD (N = 120)
Age	18 (16; 18)	17 (16;18)
Smoking (yes/no)	44 (67.7%)/21 (32.3%)	106 (88.3%)/14 (11.7%) *
Correctional facility (yes/no)	65 (29.4%)/156 (70.6%)	120 (100%)/0 (0%)
Delinquency adjudications (yes/no)	25 (38.5%)/40 (61.5%)	62 (51.7%)/58 (48.3%)
PCL-YV total scores	11 (7; 15)	27 (19; 32) *
MOAS total scores	13 (5; 20)	33 (21; 44) *

Data are represented as median and interquartile range, or as total number and frequency. CD—conduct disorder; MOAS—Modified Overt Aggression Scale; N—number of subjects; PCL-YV—Hare Psychopathy Checklist: Youth Version; * *p* < 0.001 vs. control subjects.

### 3.2. The Significant Associations Between Aggressive Behavior and COMT rs4680 and rs4818 Genotypes

#### 3.2.1. PCL-YV Scale Scores and *COMT* rs4680 and rs4818 Genotypes

The PCL-YV and MOAS scales were used to assess antisocial and aggressive behavior in subjects living in the correctional facility with or without CD.

The PCL-YV scale, which consists of four domains, was used to examine the risk of developing antisocial and psychopathic personality behavior in young people [53]. The F1 domain assesses the interpersonal dimension, which measures arrogance, deceitful behavior, and pathological lying; the F2 domain assesses the affective dimension, which includes emotional numbing and a lack of regret or empathy; F3 is the behavioral domain that assesses antisocial tendencies, irritability, impulsivity, and lack of goals; and the F4 domain assesses the criminal behavioral dimension, which includes poor anger control and criminal behavior in adolescents.

**Figure 1:**
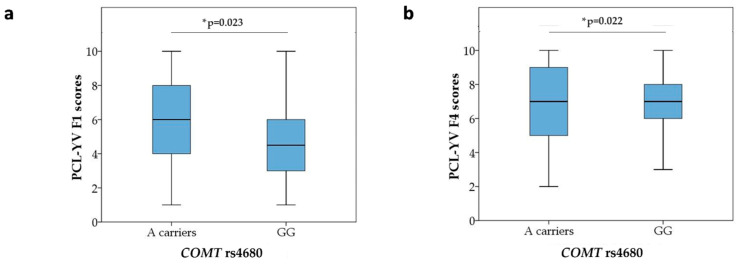
Significantly different PCL-YV scores on (**a**) interpersonal (F1) and (**b**) criminal (F4) domains in detained subjects with CD between *COMT* rs4680 A carriers and GG carriers. The data are represented as median and interquartile range. The central box represents the interquartile range, the middle line represents the median, and the vertical line extends from the minimum to the maximum value. * Mann–Whitney test *p*-value; significant *p*-value *p* < 0.025. CD—conduct disorder; dominant model—A carriers (combined AA + GA genotypes) vs. GG carriers; PCL-YV—Hare Psychopathy Checklist: Youth Version; PCL-YV F1 scores—interpersonal dimension scores; PCL-YV F4 scores—criminal behavior dimension scores.

When evaluating the dominant model with the Mann–Whitney test, *COMT* rs4680 GG carriers with CD had slightly lower PCL-YV scores than the A allele (the combined group of AA homozygotes and GA heterozygotes) carriers (*p* = 0.046), but the significance was lost after correction for multiple testing (Table 2). However, *COMT* rs4680 A carriers with CD had significantly higher scores on the PCL-YV F1 (*p* = 0.023) and F4 (*p* = 0.022) domains, which assessed the interpersonal and criminal behavior dimension, compared to GG carriers, respectively (Table 2, Figure 1). Other scores in the PCL-YV F2 and F3 domains were not significantly different among *COMT* rs4680 A and GG carriers in subjects with CD.

In a codominant model (evaluating AA, GA, and GG genotypes), the Kruskal–Wallis ANOVA revealed that the total PCL-YV scores did not differ significantly in subjects with CD or control subjects carrying *COMT* rs4680 AA, AG, and GG genotypes.

No significant differences (Mann–Whitney test) between *COMT* rs4680 A carriers compared to GG carriers (codominant model) were found in the PCL-YV total scores, or the specific scores in the PCL-YV domains (F1, F2, F3, and F4) in control subjects detained in the correctional facility (Table 2).

**Figure 2:**
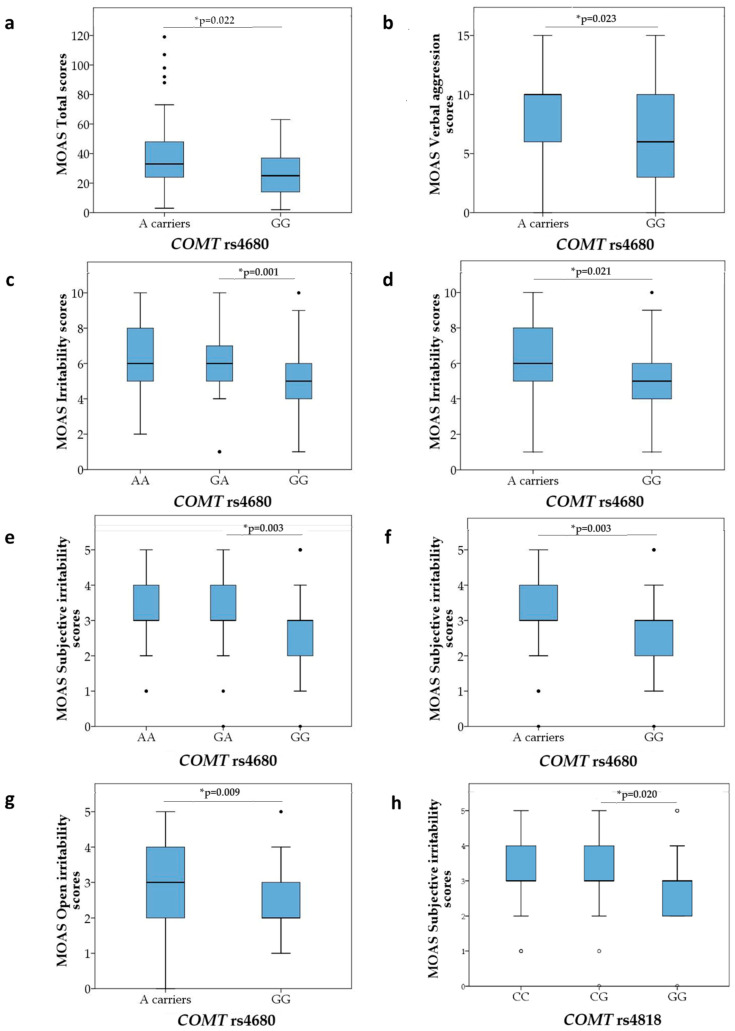
Significantly different MOAS scores in detained subjects with conduct disorder: (**a**) total scores between *COMT* rs4680 A and GG carriers; (**b**) verbal aggression scores between *COMT* rs4680 A and GG carriers; (**c**) irritability scores between *COMT* rs4680 AA, GA, and GG genotypes; (**d**) irritability scores between *COMT* rs4680 A and GG carriers; (**e**) subjective irritability scores between *COMT* rs4680 AA, GA, and GG genotypes; (**f**) subjective irritability scores between *COMT* rs4680 A and GG carriers; (**g**) open irritability scores between *COMT* rs4680 A and GG carriers; and (**h**) subjective irritability scores in subjects with CD carrying *COMT* rs4818 CC, CG, and GG genotypes. The data are presented as medians and interquartile range. The central box represents the interquartile range, the middle line represents the median, the vertical line extends from the minimum to the maximum value, while the separate dots represent the outliers. * Mann–Whitney test *p*-value (when comparing 2 groups—dominant model) or Kruskal–Wallis ANOVA *p*-value (when comparing 3 groups—codominant model). Significant *p*-value *p* < 0.025; dominant model—A carriers (combined AA + GA genotypes) vs. GG carriers; codominant model—CC, CG, and GG genotypes.

These results revealed that (1) in detained subjects with CD, *COMT* rs4680 A carriers exhibited increased severity of arrogance, deceitful behavior, pathological lying, poor anger control, and criminal behavior compared to GG carriers (Figure 1); (2) the F2 and F3 domains of the PCL-YV did not show significant associations with the *COMT* rs4680 polymorphism in detained subjects with CD; and (3) the *COMT* rs4680 polymorphism was not significantly associated with PCL-YV scores in the control detained subjects without CD.

When evaluating the association between the other *COMT* SNP, *COMT* rs4818, and PCL-YV scores, in the codominant or dominant model, the PCL-YV total scores and scores on the F1, F2, F3, and F4 domains assessing interpersonal, affective, behavioral, and criminal dimensions were not significantly different between subjects with CD subdivided into carriers of the *COMT* rs4818 CC, CG, and GG genotypes or subdivided into G and CC carriers (Kruskal–Wallis ANOVA; Table 3). A significant association (*p* = 0.004, Kruskal–Wallis ANOVA) was found in a small (N = 4) group of control subjects from the correctional facility, showing that carriers of the *COMT* rs4818 CG genotype had higher scores in the F1 = interpersonal dimension compared to GG homozygotes (*p* = 0.018, Dunn’s post hoc test) and compared to CC carriers (*p* = 0.004, Dunn’s post hoc test). In the dominant model, *COMT* rs4818 G allele carriers had significantly higher (*p* = 0.017, Mann–Whitney test) scores on the PCL-YV F1 domain compared to CC homozygotes in control subjects. However, these findings might be explained as false positive results since the number of control subjects in this domain was only four, with no subjects in the group of GG carriers.

These results suggest that (1) the *COMT* rs4818 polymorphism is not associated with the PCL-YV total and subdomain scores in subjects with CD; (2) the results in the control subjects need to be confirmed with enlarged groups (Table 3).

#### 3.2.2. The MOAS Scores and *COMT* rs4680 and rs4818 Genotypes

The MOAS scale [44,45] was used to evaluate several phenotypic aspects of aggression in young male subjects from a correctional facility. Namely, the MOAS scale can discriminate between three dimensions that evaluate aggressiveness: aggressive behavior (subdivided into verbal aggression, aggression toward objects, auto-aggression, and physical aggression toward other people), irritability (subjective and open), and suicidality.

**Figure 3:**
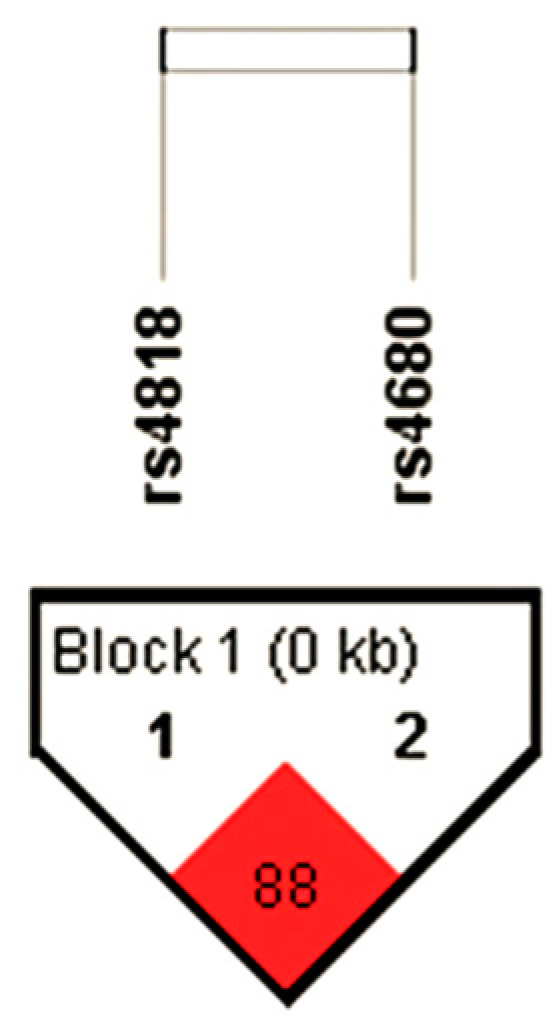
LD plot of the *COMT* rs4818 and rs4680 polymorphisms. The pairwise LD value (× 100) for the *COMT* rs4818 and rs4680 combination, as denoted in a bright red rectangle (D’ = 88), indicates a strong link between these two polymorphisms; LD—linkage disequilibrium.

In control subjects from the correctional facility, the total MOAS scores did not differ significantly between carriers of different *COMT* rs4680 genotypes or alleles when the codominant or dominant model was assessed (Table 4). In contrast, in the detained subjects with CD, in the dominant model assessed, *COMT* rs4680 A allele carriers had significantly (Mann–Whitney test) higher total MOAS scores (*p* = 0.022), verbal aggression scores (*p* = 0.023), total irritability scores (*p* = 0.003), subjective irritability scores (*p* = 0.003), and open irritability scores (*p* = 0.010) than GG homozygotes carriers (Table 4, Figure 2). In addition, slight differences, e.g., higher scores, were found in the aggression scores (*p* = 0.052) and physical aggression toward other people scores (*p* = 0.032) in the *COMT* rs4680 A allele carriers compared to GG carriers with CD (Mann–Whitney test, Table 4). This finding was confirmed in the codominant model (Kruskal–Wallis ANOVA) where *COMT* rs4680 GA heterozygotes had significantly higher MOAS total irritability scores (*p* = 0.001; Dunn’s post hoc test) and subjective irritability scores (*p* = 0.003; Dunn’s post hoc test) in comparison to the GG carriers (Table 4, Figure 3). No significant associations were observed between the MOAS auto-aggression and suicidal behavior scores and *COMT* rs4680 genotypes (Table 4).

These results revealed that (1) total aggression, verbal aggression, total irritability, subjective irritability, and open irritability, evaluated with the MOAS, were significantly associated with the *COMT* rs4680 A allele (Table 4, Figure 2).

Regarding the link between *COMT* rs4818 and aggression, subjective irritability scores in the MOAS were significantly lower in subjects with CD from the correctional facility who were carriers of the *COMT* rs4818 GG genotypes compared to the GC heterozygotes (*p* = 0.020, Dunn’s post hoc test; Table 5, Figure 2), i.e., in the codominant model. However, this effect was not detected in the dominant model (G vs. CC carriers of the *COMT* rs4818) as there was no significant (Mann–Whitney test) difference in the subjective irritability scores between *COMT* rs4818 G and CC carriers. Other domains of the MOAS were not significantly associated with the *COMT* rs4818 polymorphism. The scores in the MOAS verbal aggression, physical aggression toward objects and physical aggression toward others, auto-aggression, suicidality, irritability, and open irritability domains did not differ significantly (Kruskal–Wallis ANOVA) in control subjects subdivided into those carrying the *COMT* rs4818 CC, CG, and GG genotypes (Table 5).

These results revealed that (1) only the GG genotype of the *COMT* rs4818 was associated with reduced irritability, while the GC genotype was associated with increased subjective irritability assessed by the MOAS in detained subjects with CD (Table 5, Figure 2).

#### 3.2.3. The Lack of Association of *COMT* rs4680 and rs4818 Genotypes and Their Haplotypes with CD, Living in the Correctional Facility, Delinquent Behavior, or Smoking

The distribution of the *COMT* rs4680 and rs4818 genotypes and alleles was evaluated between subjects with CD and control subjects, but also in subjects divided by their stay in the correctional facility, having delinquency adjudications, and based on the smoking status (Supplementary Table S1).

There was no significant difference in the distribution of the *COMT* rs4680 genotypes or alleles in different diagnostic groups, i.e., in subjects with or without CD, in adolescents being situated in or out of the correctional facility, or in adolescents in the correctional facility with or without a history of convictions (Supplementary Table S1). The control subjects out of detention had a similar distribution of *COMT* rs4680 genotypes (codominant model assessing AA, GA, and GG genotypes: χ^2^ = 1.798; *p* = 0.407; dominant model assessing A vs. GG carriers: χ^2^ = 0.037; *p* = 0.847) as the control subjects living in the correctional facility. Adolescents who were smokers had a similar distribution of *COMT* rs4680 genotypes and alleles as non-smokers (χ^2^-test; Supplementary Table S1).

The distribution (χ^2^-test) of the *COMT* rs4818 genotypes differed nominally between subjects with CD and control subjects (χ^2^ = 7.274; *p* = 0.026), but after *p*-value correction, this was not statistically significant. Specifically, *COMT* rs4818 GG carriers were more prevalent in the CD group (55.3%; R = 2.0) compared to the control group (44.7%; R = −1.7). In addition, similar frequencies of the *COMT* rs4818 genotypes and alleles were detected in adolescents in and out of the correctional facility, with or without delinquency adjudications, and between smokers and non-smokers in the codominant (assessing CC, CG, and GG genotypes) or dominant (assessing G vs. CC carriers) model (χ^2^; Supplementary Table S2).

These results collectively suggest (1) a similar distribution of the *COMT* rs4680 and rs4818 genotypes or alleles between adolescents living in or out of detention, or within adolescents in a correctional facility, subdivided into those with or without delinquency adjudications or between smokers and non-smokers.

The haplotype analysis showed strong linkage disequilibrium (LD) between *COMT* rs4818 and rs4680 (D’ × 100 = 88) (Figure 3); therefore, the haplotypes for the *COMT* rs4818-rs4680 block were determined for each individual using an expectation–maximization algorithm. The most prevalent haplotype in the total sample was CA (48.3%), followed by GG (35.2%) and CG (14.2%). The least common haplotype was GA (2.3%), which was excluded from the further analyses due to its low frequency.

The frequency of *COMT* rs4818-rs4680 haplotypes (CA, GG, and CG) was not significantly different (χ^2^-test) between subjects divided depending on the CD diagnosis, correctional facility confinement, delinquency adjudications, and smoking status (Supplementary Table S3).

When subjects were subdivided according to the PCL-YV total scores or F1, F2, F3, and F4 subdomain scores, no significant (Kruskal–Wallis ANOVA) differences in the scores related to the total PCL-YV or subdomains (F1, F2, F3, and F4) scores were found in subjects with CD, as well as in control subjects, carriers of the *COMT* rs4818-rs4680 CA, GG, and CG haplotypes, from the correctional facility (Supplementary Table S4).

No significant associations (Kruskal–Wallis ANOVA) of the *COMT* rs4818-rs4680 CA, GG, and CG haplotypes with the MOAS total, verbal aggression, physical aggression toward objects and physical aggression toward others, auto-aggression, suicidality, irritability, subjective irritability, and open irritability domain scores were found either in control subjects or participants with CD detained in the correctional facility (Supplementary Table S5).

These results showed that (1) *COMT* rs4818-rs4680 haplotypes (CA, GG, and CG) were not significantly associated with CD, living in the correctional facility, delinquency adjudications, and smoking status; (2) in detained adolescents, *COMT* rs4818-rs4680 haplotypes were not significantly associated with PCL-YV total or subdomain (F1, F2, F3, and F4) scores; and (3) *COMT* rs4818-rs4680 haplotypes were not significantly associated with the MOAS total and subscale scores in adolescents living in the correctional facility.

## 4. Discussion

The main findings of this study are that in detained adolescents with CD, (1) carrying one or two A alleles of the *COMT* rs4680 polymorphism is associated with aggressive behavior, especially with higher levels of irritability, both subjective and open, as well as verbal aggression, (2) *COMT* rs4680 A allele carriers, compared to GG homozygotes, had more prominent psychopathic and antisocial traits, especially pathological lying and manipulative and criminal behavior, and (3) *COMT* rs4818 GC genotype carriers demonstrated higher subjective irritability compared to GG homozygotes. These results collectively suggest an association between lower *COMT* activity alleles and aggressive traits in detained male adolescents with CD. No significant association of the *COMT* rs4680 and rs4818 genotypes, or rs4680-rs4818 haplotypes, was observed with the risk of developing CD, detention in a correctional facility, smoking status, or history of delinquency adjudications. Both the *COMT* rs4680 and rs4818 polymorphisms were not associated with the severity of aggressive symptoms in control subjects without CD, who exhibited some level of aggression and were detained in the correctional facility. Moreover, *COMT* rs4680-rs4818 haplotypes were not related to aggressive traits in detained adolescents. The novel aspect of this study is the evaluation of the association between *COMT* rs4818 genotypes and *COMT* rs4680-rs4818 haplotypes with symptoms of aggression (assessed using PCL-YV and MOAS) in adolescents living in the correctional facility with CD.

Genetic studies of aggression mostly focused on the genes regulating dopamine and serotonin metabolism and signaling and neuroendocrine functions [54]. The dysfunction of the PFC and dopamine-mediated cognitive functions [23] has been associated with problematic and antisocial behavior in children, youth, and adults [4,55,56]. Therefore, *COMT* rs4680, which modulates dopamine function and affects aggressive behavior in youth, is frequently studied [4,18,37,38], but with inconsistent findings [4], while reports regarding *COMT* rs4818, which also affects dopamine activity and might influence aggressive behavior, remain limited [39,55].

In our study, *COMT* rs4680 A allele carriers had more severe psychopathic and antisocial traits, assessed with the PCL-YV, compared to the carriers of the GG genotype. In line with this finding, the *COMT* rs4680 A allele has been previously associated with physical violence against others and angry behavior in patients with schizophrenia [57,58], physical and relational violence in young adults [38], higher hyperactivity, impulsivity, and inattentive symptoms in children with ADHD [48], higher aggressive and depressive symptoms and a history of suicide attempts in male subjects with alcoholism [59], and with higher scores on difficulties in abstract thinking in patients with schizophrenia [60] compared to GG homozygotes. In contrast to our data, the *COMT* rs4680 GG genotype was linked to aggressive symptoms of CD in young people with ADHD [61], or with more violent behavior and an increased risk of misconduct in children with ADHD [62], or with the risk of more severe symptoms of CD in male adolescent delinquents with ADHD confined in the correctional facility [63]. The differences between studies are that our subjects did not have comorbid ADHD. In line with the association of the *COMT* rs4680 A allele with antisocial and criminal behavior and irritability in adolescents with CD, Iraqi prisoners carrying one or two *COMT* rs4680 A alleles had a significantly higher risk of criminal behavior and committed more violent crimes than GG carriers [64].

As a result of the low suicidal and auto-aggression scores in the majority of detained adolescents with CD and the control group, the present study found no association between *COMT* rs4680 or rs4818 and suicidal behavior.

In agreement with data suggesting no significant effect of the *COMT* rs4818 genotypes on domestic violence in adult Chinese alcoholics [55], *COMT* rs4818 was not significantly associated with violence, aggression, psychopathic and antisocial traits, or criminal behavior, assessed using the PCL-YV total and subdomain scores, in the detained adolescents with CD.

However, both *COMT* SNPs were related to MOAS scores. Namely, adolescents with CD, carriers of the *COMT* rs4680 A allele, had higher scores on total MOAS, verbal aggression, and total, subjective, and open irritability compared to GG homozygotes, while *COMT* rs4818 GC carriers had higher MOAS subjective irritability scores than GG carriers. No studies have evaluated a link between *COMT* rs4818 and aggression in detained adolescents with CD, and therefore we cannot interpret these results in light of existing data. Comparably to our data, in predominately younger aggressive boys with ADHD, the *COMT* rs4818 GC genotype was over-represented [39], and this genotype was nominally related to the CU traits [39], while in adult males with schizophrenia, *COMT* rs4818 CC homozygotes had more severe difficulties in abstract thinking compared to G allele carriers [60].

In our study, *COMT* rs4818–rs4680 haplotypes were not related to CD in adolescents living in the correctional facility or to other aggression-related symptoms assessed using PCL-YV or MOAS. Diverse findings were reported in adult patients with schizophrenia, where the *COMT* rs4818-rs4680 GA haplotype was related to the highest scores of somatic concerns in male patients [60], while the rs4818-rs4680 GG haplotype was associated with elevated scores in negative symptoms and anhedonia in female patients [31]. Different diagnoses (schizophrenia vs. CD), sex (both sexes vs. males), age period (adults vs. youth), and different symptoms and assessments might explain divergent findings between these [31,60] and the present study.

Evidence suggests that *COMT* may interact with traumatic and adverse events in childhood, which might predict aggression later on in life [65]. When analyzing gene × environment (GxE) interactions between the *COMT* rs4680 polymorphism and serious life events on childhood aggression, it was reported that children carrying the GG genotype, who were exposed to serious life events, exhibited more aggression than the A carriers [66]. However, in a supportive environment, COMT rs4680 GG carriers exhibited a lower level of aggression compared to the A carriers [66], implicating that although the *COMT* rs4680 A allele is usually associated with higher aggression, it is less affected by environmental factors (socioeconomic status and exposure to serious life events, early caregiving environment and parenting styles, prenatal maternal smoking, or low birth weight) [4]. Therefore, the association of the *COMT* rs4680 polymorphism with psychopathy-related behaviors is affected by GxE interactions [4], and thus we have evaluated the GxE interaction. However, the environment, i.e., living in the correctional facility, was not associated with *COMT* rs4680 or rs4818 genotypes or haplotypes, as the frequency of the *COMT* rs4680 and rs4818 alleles, genotypes, and haplotypes did not differ in male adolescents living in their homes or in the correctional facility. This absence of a significant GxE interaction might be attributable to the difference between prisons [64] and a correctional facility (present study), as, in Croatia, a correctional facility is used to rehabilitate adolescents and correct their pathological behaviors, while in prison, people who have committed crimes serve their sentence.

Genetic and environmental influences on CD and the development of antisocial personality disorders across the lifespan were found, with males exhibiting more pronounced symptoms than females [67]. Moreover, COMT demonstrated sexual dimorphism in psychiatric disorders, and there are sex-related differences in cognitive performance and brain functions, probably due to the estrogen cycle [68] as disruptive behavior problems and CD are significantly more common in males than females [1,2]. Therefore, we controlled for this effect by including only male subjects. Further studies should investigate the effects of *COMT* rs4680 or rs4818 polymorphisms in female subjects with CD and different subtypes of aggressive behavior, as well as in adults with antisocial personality disorders, including other potential moderating environmental factors.

In general, genetic findings of the candidate genes related to aggression in youth are affected by moderate sample sizes, ethnic and age- and sex-related differences, and different methods of evaluating aggressive behavior, and these confounders result in inconclusive findings of the main effects of genes, gene–gene interactions, and GxE interactions [18]. Although high alcohol use and probable dependence, associated with increased aggressive behavior, are found in the criminal justice system [69], in our study, current substance use disorder was an exclusion criterion, and our adolescents were living in the correctional facility, and before being included in this study, they were not using any drugs or medications and did not have a diagnosis of substance use disorder.

One limitation of our study is that we evaluated only two polymorphisms in the *COMT* gene. However, these two *COMT* SNPs are functional, affecting *COMT* activity. The second limitation is that, due to the study protocol and sampling in the correctional facility for male youth, we could not include female adolescents.

The strengths of this study include the participation of 341 male Caucasian adolescents, 16 to 18 years old (120 with a diagnosis of CD and 221 without CD), in the diagnoses of CD performed by child psychiatrists and psychologists, as well as the detailed evaluation of aggression with the PCL-YV, MOAS, and SNAP-IV. Another strength lies in the control of the association of *COMT* rs4680 and rs4818 genotypes and haplotypes with aggressive behavior for possible confounders, such as age, sex, smoking, and environment.

## 5. Conclusions

In brief, *COMT* rs4680 A allele carriers had higher levels of aggressive behavior (subjective and open irritability, verbal aggression, aggression toward objects, and psychopathic and antisocial traits, especially pathological lying, manipulative and criminal behavior), while carriers of the *COMT* rs4818 GC genotype demonstrated higher subjective irritability compared to G allele carriers, in detained youth with CD. These results suggest that the presence of the *COMT* alleles, related to lower COMT activity, is associated with increased aggressive traits in adolescents. Further research is needed to confirm the association between *COMT* rs4680 and rs4818 polymorphisms and the risk of psychopathy-related behaviors in youth, adults, male and female populations, and in different ethnicities, but also in other personality disorders.

## Figures and Tables

**Table 2:** The PCL-YV total scores as well as scores on interpersonal (F1), affective (F2), behavioral (F3), and criminal (F4) domains in subjects with CD and control subjects from the correctional facility carrying various *COMT* rs4680 genotypes and alleles.

PCL-YV Scores	Group	Codominant Model COMT rs4680			Statistics	Dominant Model COMT rs4680		Statistics
		AA	GA	GG		A	GG	
Total scores	CD	28 (19; 34)	27 (21; 32)	23 (17; 30)	H = 4.40; *p* = 0.111	27 (20; 32)	23 (17; 30)	U = 1119.0; *p* = 0.046
	Control	10 (7; 15)	12 (9; 16)	10 (8; 15)	H = 1.57; *p* = 0.457	11 (7; 15)	10 (8; 15)	U = 312.0; *p* = 0.669
F1 domain scores	CD	6 (4; 8)	6 (4; 8)	5 (3; 6)	H = 5.19; *p* = 0.075	6 (4; 8)	5 (3; 6)	U = 1074.0; ***p* = 0.023**
	Control	1 (0; 2)	2 (1; 4)	1 (0; 3)	H = 4.21; *p* = 0.122	2 (1; 3)	1 (0; 3)	U = 292.5; *p* = 0.447
F2 domain scores	CD	8 (6; 10)	8 (6; 9)	6 (4; 9)	H = 3.88; *p* = 0.144	8 (6; 10)	6 (4; 9)	U = 1142.0; *p* = 0.059
	Control	4 (2; 5)	3 (2; 5)	3 (2; 4)	H = 0.55; *p* = 0.760	4 (2; 5)	3 (2; 4)	U = 293.5; *p* = 0.461
F3 domain scores	CD	7 (4; 9)	6 (5; 8)	5 (3; 7)	H = 3.93; *p* = 0.140	7 (4; 8)	5 (3; 7)	U = 1132.5; *p* = 0.053
	Control	2 (0; 4)	2 (2; 5)	3 (2; 5)	H = 0.27; *p* = 0.874	2 (1; 4)	3 (2; 5)	U = 321.0; *p* = 0.777
F4 domain scores	CD	8 (6; 10)	7 (5; 9)	7 (6; 8)	H = 5.42; *p* = 0.066	7 (5; 9)	7 (6; 8)	U = 1068.0; ***p* = 0.022**
	Control	3 (1; 4)	3 (2; 4)	3 (2; 4)	H = 0.61; *p* = 0.739	3 (2; 4)	3 (2; 4)	U = 337.5; *p* = 0.993

The data are presented as median and interquartile range, while significant *p*-values (Mann–Whitney test) are denoted in bold. CD—conduct disorder; codominant model—AA, GA, and GG genotypes; dominant model—A carriers (combined AA + GA genotypes) vs. GG carriers; F1 domain—interpersonal domain scores of the PCL-YV; F2 domain—affective domain scores of the PCL-YV; F3 domain—behavioral domain scores of the PCL-YV; F4 domain—criminal domain scores of the PCL-YV; PCL-YV—Hare Psychopathy Checklist: Youth Version.

**Table 3:** The PCL-YV total scores as well as scores on interpersonal (F1), affective (F2), behavioral (F3), and criminal (F4) domains in subjects with CD and control subjects from the correctional facility carrying various *COMT* rs4818 genotypes and alleles.

PCL-YV Scores	Group	Codominant Model *COMT* rs4818			Statistics	Dominant Model *COMT* rs4818		Statistics
		CC	CG	GG		G	CC	
Total scores	CD	24 (18; 28)	26 (19; 32)	25 (18; 30)	H = 1.23; *p* = 0.539	26 (19; 32)	24 (18; 28)	U = 907.5; *p* = 0.507
	Control	10 (7; 15)	12 (11; 15)	10 (7; 13)	H = 2.99; *p* = 0.224	12 (10; 15)	10 (7; 15)	U = 274.0; *p* = 0.185
F1 domain scores	CD	5 (4; 7)	5 (4; 7)	5 (3; 8)	H = 0.07; *p* = 0.967	5 (4; 7)	5 (4; 7)	U = 959.0; *p* = 0.802
	Control	1 (0; 2)	3 (1; 5)	0 (0; 2)	H = 11.23; ***p* = 0.004**	2 (1; 3)	1 (0; 2)	U = 217.5; ***p* = 0.017**
F2 domain scores	CD	6 (5; 8)	8 (4; 10)	6 (5; 9)	H = 2.42; *p* = 0.298	7 (5; 10)	6 (5; 8)	U = 851.5; *p* = 0.262
	Control	4 (2; 5)	3 (2; 5)	3 (3; 4)	H = 0.15; *p* = 0.927	3 (2; 4)	4 (2; 5)	U = 326.5; *p* = 0.697
F3 domain scores	CD	6 (4; 7)	7 (4; 9)	4 (4; 6)	H = 4.31; *p* = 0.116	6 (4; 8)	6 (4; 7)	U = 964.5; *p* = 0.836
	Control	3 (1; 6)	3 (2; 4)	2 (1; 4)	H = 0.36; *p* = 0.836	2 (2; 4)	3 (1; 6)	U = 327.5; *p* = 0.709
F4 domain scores	CD	6 (5; 8)	7 (5; 8)	8 (6; 8)	H = 0.55; *p* = 0.758	7 (5; 8)	6 (5; 8)	U = 912.5; *p* = 0.529
	Control	3 (1; 4)	3 (2; 4)	4 (2; 5)	H = 1.69; *p* = 0.429	3 (2; 5)	3 (1; 4)	U = 277.5; *p* = 0.200

The data are represented as the median and interquartile range, while significant *p*-values are denoted in bold: Kruskal–Wallis ANOVA; *p* = 0.004 between CG and GG carriers; Mann–Whitney test; *p* = 0.017 between G and CC carriers in control subjects. CD—conduct disorder; codominant model—CC, CG, and GG genotypes; dominant model—G carriers (combined GG + GC genotypes) vs. CC carriers; F1 domain—interpersonal domain scores of the PCL-YV; F2 domain—affective domain scores of the PCL-YV; F3 domain—behavioral domain scores of the PCL-YV; F4 domain—criminal domain scores of the PCL-YV; PCL-YV—Hare Psychopathy Checklist: Youth Version.

**Table 4:** The MOAS total scores and scores on aggression, suicidality, and irritability domains in subjects with CD and control subjects from the correctional facility carrying various *COMT* rs4680 genotypes and alleles.

MOAS Scores	Group	Codominant Model *COMT* rs4680			Statistics	Dominant Model *COMT* rs4680		Statistics
		AA	GA	GG		A	GG	
Total scores	CD	32 (22; 48)	35 (25; 47)	25 (14; 37)	H = 5.42; *p* = 0.066	33 (24; 48)	25 (14; 37)	U = 1068.0; *p* = 0.022
	Control	9 (5; 18)	12 (4; 22)	18 (10; 23)	H = 1.69; *p* = 0.429	12 (5; 20)	18 (10; 23)	U = 273.0; *p* = 0.286
Aggression (total) scores	CD	26 (15; 37)	27 (20; 40)	21 (8; 31)	H = 3.91; *p* = 0.141	27 (17; 40)	21 (8; 31)	U = 1129.0; *p* = 0.052
	Control	5 (2; 11)	9 (1; 17)	12 (6; 17)	H = 0.92; *p* = 0.632	8 (1; 12)	12 (6; 17)	U = 284.0; *p* = 0.374
*Verbal* *aggression* scores	CD	9 (6; 15)	10 (6; 10)	6 (3; 10)	H = 5.30; *p* = 0.071	10 (6; 10)	6 (3; 10)	U = 1078.5; *p* = 0.023
	Control	3 (1; 6)	3 (1; 7)	6 (1; 7)	H = 1.24; *p* = 0.538	3 (1; 7)	6 (1; 7)	U = 282.5; *p* = 0.356
*Physical* *aggression* *scores* *toward* *objects*	CD	6 (2; 10)	4 (2; 12)	2 (2; 6)	H = 4.14; *p* = 0.126	6 (2; 12)	2 (2; 6)	U = 1180.5; *p* = 0.095
	Control	2 (0; 4)	2 (0; 2)	2 (0; 2)	H = 0.75; *p* = 0.688	2 (0; 3)	2 (0; 2)	U = 295.0; *p* = 0.452
*Auto-* *aggression* scores	CD	0 (0; 6)	0 (0; 6)	0 (0; 6)	H = 0.18; *p* = 0.916	0 (0; 6)	0 (0; 6)	U = 1418.5; *p* = 0.864
	Control	0 (0; 0)	0 (0; 0)	0 (0; 0)	H = 0.66; *p* = 0.719	0 (0; 0)	0 (0; 0)	U = 313.5; *p* = 0.552
*Physical* *aggression* *scores* *toward* *others*	CD	9 (3; 9)	9 (3; 11)	6 (3; 9)	H = 5.59; *p* = 0.061	9 (3; 9)	6 (3; 9)	U = 1109.5; *p* = 0.032
	Control	0 (0; 3)	2 (0; 3)	3 (3; 3)	H = 3.44; *p* = 0.179	2 (0; 3)	3 (3; 3)	U = 236.0; *p* = 0.074
Irritability (total) scores	CD	6 (5; 8)	7 (6; 8)	5 (4; 6)	H = 11.44; ***p* = 0.003**	6 (5; 8)	5 (4; 6)	U = 891.5; *p* = 0.001
	Control	4 (2; 5)	4 (2; 6)	4 (3; 6)	H = 1.12; *p* = 0.571	4 (2; 6)	4 (3; 6)	U = 301.0; *p* = 0.540
*Subjective* *irritability* *scores*	CD	3 (3; 4)	3 (3; 4)	3 (2; 3)	H = 9.26; ***p* = 0.010**	3 (3; 4)	3 (2; 3)	U = 970.0; *p* = 0.003
	Control	2 (1; 3)	2 (1; 3)	3 (3; 3)	H = 2.11; *p* = 0.348	2 (1; 3)	3 (3; 3)	U = 265.0; *p* = 0.215
*Open* *irritability* *scores*	CD	3 (2; 4)	3 (2; 4)	2 (2; 3)	H = 6.91; *p* = 0.032	3 (2; 4)	2 (2; 3)	U = 1026.5; *p* = 0.009
	Control	1 (1; 2)	1 (1; 2)	1 (1; 3)	H = 1.01; *p* = 0.603	1 (1; 2)	1 (1; 3)	U = 321.5; *p* = 0.774
Suicidality (total) scores	CD	1 (0; 2)	1 (0; 1)	1 (0; 1)	H = 2.09; *p* = 0.352	1 (0; 2)	1 (0; 1)	U = 1351.0; *p* = 0.490
	Control	0 (0; 1)	1 (0; 1)	0 (0; 1)	H = 1.80; *p* = 0.407	1 (0; 1)	0 (0; 1)	U = 279.5; *p* = 0.291

The data are represented as median and interquartile range, while significant *p*-values are denoted in bold. Significant differences (Kruskal–Wallis ANOVA) between *COMT* rs4680 GA heterozygotes and GG carriers in the MOAS total irritability scores (*p* = 0.001; Dunn’s post hoc test) and subjective irritability scores (*p* = 0.003; Dunn’s post hoc test) and between *COMT* rs4680 A allele carriers and GG homozygotes carriers (Mann–Whitney test) in the total MOAS scores (*p* = 0.022), verbal aggression scores (*p* = 0.023), total irritability scores (*p* = 0.003), subjective irritability scores (*p* = 0.003), and open irritability scores (*p* = 0.010) in the detained subjects with CD; codominant model–AA, GA, and GG genotypes; CD—conduct disorder; dominant model—A carriers (combined AA + GA genotypes) vs. GG carriers; MOAS—Modified Overt Aggression Scale.

**Table 5:** MOAS total scores and scores on aggression, suicidality, and irritability domains in subjects with CD and control subjects from the correctional facility carrying various *COMT* rs4818 genotypes and alleles.

MOAS Scores	Group	Codominant Model *COMT* rs4818			Statistics	Dominant Model *COMT* rs4818		Statistics
		CC	CG	GG		G	CC	
Total scores	CD	33 (19; 42)	32 (22; 46)	21 (13; 37)	H = 3.55; *p* = 0.169	32 (19; 39)	33 (19; 42)	U = 912.0; *p* = 0.531
	Control	14 (4; 20)	12 (6; 20)	21 (18; 25)	H = 2.34; *p* = 0.311	16 (6; 23)	14 (4; 20)	U = 310.5; *p* = 0.502
Aggression (total) scores	CD	27 (14; 35)	24 (16; 34)	16 (8; 30)	H = 2.50; *p* = 0.286	23 (14; 32)	27 (14; 35)	U = 909.5; *p* = 0.518
	Control	9 (1; 15)	9 (4; 12)	12 (11; 15)	H = 1.45; *p* = 0.484	9 (4; 12)	9 (1; 15)	U = 312.0; *p* = 0.518
*Verbal* *aggression* scores	CD	10 (6; 10)	7 (5; 10)	5 (3; 10)	H = 4.91; *p* = 0.086	7 (5; 10)	10 (6; 10)	U = 838.0; *p* = 0.215
	Control	4 (1; 6)	3 (1; 7)	7 (6; 7)	H = 2.96; *p* = 0.227	4 (1; 7)	4 (1; 6)	U = 302.5; *p* = 0.410
*Physical* *aggression* *scores* *toward* *objects*	CD	6 (2; 10)	4 (2; 8)	2 (2; 6)	H = 1.26; *p* = 0.532	3 (2; 8)	6 (2; 10)	U = 868.0; *p* = 0.319
	Control	0 (0; 3)	2 (0;2)	2 (1; 2)	H = 1.58; *p* = 0.454	2 (0; 2)	0 (0; 3)	U = 285.5; *p* = 0.235
*Auto-* *aggression* scores	CD	0 (0; 8)	3 (0; 6)	0 (0; 6)	H = 1.57; *p* = 0.457	0 (0; 6)	0 (0; 8)	U = 924.5; *p* = 0.750
	Control	0 (0; 0)	0 (0; 0)	0 (0; 3)	H = 0.05; *p* = 0.975	0 (0; 0)	0 (0; 0)	U = 347.5; *p* = 0.990
*Physical* *aggression* *scores* *toward* *others*	CD	9 (3; 9)	9 (3; 9)	3 (3; 9)	H = 1.05; *p* = 0.592	8 (3; 9)	9 (3; 9)	U = 970.5; *p* = 0.870
	Control	3 (0; 3)	3 (0; 3)	3 (3; 3)	H = 1.20; *p* = 0.548	3 (0; 3)	3 (0; 3)	U = 322.5; *p* = 0.627
Irritability (total) scores	CD	6 (5; 7)	7 (5; 8)	5 (4; 6)	H = 5.95; *p* = 0.051	6 (5; 7)	6 (5; 7)	U = 983.5; *p* = 0.958
	Control	4 (2; 6)	4 (2; 6)	6 (5; 6)	H = 2.56; *p* = 0.278	4 (2; 6)	4 (2; 6)	U = 320.5; *p* = 0.620
*Subjective* *irritability* *scores*	CD	3 (3; 4)	3 (3; 4)	3 (2; 3)	H = 7.87; ***p* = 0.020**	3 (3; 4)	3 (3; 4)	U = 889.5; *p* = 0.395
	Control	3 (1; 3)	2 (1; 3)	3 (3; 4)	H = 2.42; *p* = 0.298	3 (1; 3)	3 (1; 3)	U = 335.0; *p* = 0.810
*Open* *irritability* scores	CD	3 (2; 4)	3 (2; 4)	3 (2; 3)	H = 2.59; *p* = 0.274	3 (2; 4)	3 (2; 4)	U = 886.0; *p* = 0.387
	Control	1 (1; 3)	1 (1; 3)	3 (2; 3)	H = 1.65; *p* = 0.438	1 (1; 3)	1 (1; 3)	U = 308.0; *p* = 0.448
Suicidality (total) scores	CD	1 (0; 1)	1 (0; 2)	1 (0; 1)	H = 0.86; *p* = 0.650	1 (0; 2)	1 (0; 1)	U = 986.0; *p* = 0.973
	Control	1 (0; 1)	0 (0; 1)	1 (0; 7)	H = 0.44; *p* = 0.802	0 (0; 1)	1 (0; 1)	U = 321.0; *p* = 0.600

The data are represented as median and interquartile range, while significant *p*-values are denoted in bold. A significant difference was found among subjects with CD: Kruskal–Wallis ANOVA; *p* = 0.020 between GG carriers and GC carriers; codominant model—CC, CG, and GG genotypes; CD—conduct disorder; dominant model—G carriers (combined GG + GC genotypes) vs. CC carriers; MOAS—Modified Overt Aggression Scale.

## Data Availability

The original contributions presented in this study are included in the article/Supplementary Material. Further inquiries can be directed to the corresponding author.

## Supplementary Materials

The following supporting information can be downloaded at: http://www.mdpi.com/article/10.3390/biom15040554/s1, Table S1: The lack of association between the *COMT* rs4680 polymorphism with CD, delinquent behavior or smoking; Table S2. The lack of association of the COMT rs4818 polymorphism with CD, delinquent behavior, or smoking; Table S3. The lack of association between COMT rs4818-rs4680 CA, GG, and CG haplotypes with CD, delinquent behavior, or smoking; Table S4. The PCL-YV total scores as well as scores on interpersonal (F1), affective (F2), behavioral (F3), and criminal (F4) domains in participants with CD and control subjects from the correctional facility carrying COMT rs4818-rs4680 CA, GG, and CG haplotypes; Table S5. The MOAS total scores and scores on total aggression, verbal aggression, physical aggression toward objects and physical aggression toward others, auto-aggression, suicidality, irritability, and subjective and open irritability domains in participants with CD and control subjects from correctional facility carrying various COMT rs4818-rs4680 CA, GG, and CG haplotypes.

## Abbreviations

The following abbreviations are used in this manuscript:

ADHD	attention deficit hyperactivity disorder
CD	conduct disorder
COMT	catechol-O-methyl transferase
CU	callous–unemotional trait
MOAS	Modified Overt Aggression Scale
PFC	prefrontal cortex
PCL-IV	Hare Psychopathy Checklist: Youth Version
SNAP-IV	Swanson, Nolan and Pelham Questionnaire
