# Supplementary material for: Association of Lower COMT Activity Alleles with Aggressive Traits in Male Youth with Conduct Disorder Living in a Correctional Facility
Source: Biomolecules. 2025 Apr 9;15(4):554. doi: 10.3390/biom15040554 (PMC12024663; doi:10.3390/biom15040554)
Supplement: Supplementary file 1 [file biomolecules-15-00554-s001.zip › biomolecules-3475157-supplementary.pdf]

Supplementary Table S1. The lack of association between the *COMT* rs4680 polymorphism with CD, delinquent behavior or smoking

COMT **rs4680**	**Group**	**Codominant model**			**Dominant model**	
		**AA**	**GA**	**GG**	**A**	**GG**
Diagnostic group	CD	58 (65.9%)	117 (67.6%)	46 (57.5%)	86 (33.0%)	34 (42.5%)
	Control	30 (34.1%)	56 (32.4%)	34 (42.5%)	175 (67.0%)	46 (57.5%)
	Statistics	χ^2=2.524; p=0.283			χ^2=2.449; p=0.118	
Correctional facility	Yes	51 (27.6%)	87 (47.0%)	47 (25.4%)	138 (74.6%)	47 (25.4%)
	No	37 (23.7%)	86 (55.1%)	33 (21.2%)	123 (78.8%)	33 (21.2%)
	Statistics	χ^2=2.233; p=0.327			χ^2=0.852; p=0.356	
Delinquency adjudications	Yes	24 (47.1%)	43 (49.4%)	20 (42.6%)	67 (48.6%)	20 (42.6%)
	No	27 (52.9%)	44 (50.6%)	27 (57.4%)	71 (51.4%)	27 (57.4%)
	Statistics	χ^2=0.579; p=0.749			χ^2=0.506; p=0.477	
Smoking	Yes	43 (84.3%)	68 (78.2%)	39 (83.0%)	111 (80.4%)	39 (83.0%)
	No	8 (15.7%)	19 (21.8%)	8 (17.0%)	27 (19.6%)	8 (17.0%)
	Statistics	χ^2=0.941; p=0.625			χ^2=0.148; p=0.701	

The data are represented as numbers and frequencies. Codominant model – AA, AG and GG genotypes; dominant model: A carriers (combined AA+GA genotypes) vs. GG genotype carriers.

Supplementary Table S2. The lack of association of the *COMT* rs4818 polymorphism with CD, delinquent behavior or smoking

COMT **rs4818**	**Group**	**Codominant model**			**Dominant model**	
		**CC**	**CG**	**GG**	**G**	**CC**
Diagnostic group	CD	33 (32.0%)	39 (33.1%)	21 (55.3%)	60 (38.5%)	33 (32.0%)
	Control	70 (68.0%)	79 (66.9%)	17 (44.7%)	96 (61.5%)	70 (68.0%)
	Statistics	χ^2=7.274; p=0.026			χ^2=1.112; p=0.292	
Correctional facility	Yes	57 (55.3%)	64 (54.2%)	25 (65.8%)	89 (57.1%)	57 (55.3%)
	No	46 (44.7%)	54 (45.8%)	13 (34.2%)	67 (42.9%)	46 (44.7%)
	Statistics	χ^2=1.634; p=0.442			χ^2=0.074; p=0.786	
Delinquency adjudications	Yes	28 (49.1%)	29 (45.3%)	13 (52.0%)	42 (47.2%)	28 (49.1%)
	No	29 (50.9%)	35 (54.7%)	12 (48.0%)	47 (52.8%)	29 (50.9%)
	Statistics	χ^2=0.374; p=0.829			χ^2=0.052; p=0.820	
Smoking	Yes	46 (80.7%)	51 (79.7%)	21 (84.0%)	72 (80.9%)	46 (80.7%)
	No	11 (19.3%)	13 (20.3%)	4 (16.0%)	17 (19.1%)	11 (19.3%)
	Statistics	χ^2=0.217; p=0.897			χ^2=0.001; p=0.976	

The data are represented as numbers and frequencies. Codominant model: CC, CG and GG genotype carriers; dominant model: G carriers (GG+CG) vs. CC genotype carriers of the *COMT* rs4818 polymorphism.

1.

Supplementary Table S3. The lack of association between the *COMT* rs4818-rs4680 CA, GG and CG haplotypes with CD, delinquent behavior or smoking.

		COMT **rs4818-rs4680 haplotypes**		
		**CA**	**GG**	**CG**
Diagnostic group	CD	79 (31.3%)	78 (42.4%)	26 (36.1%)
	Control	173 (68.7%)	106 (57.6%)	46 (63.9%)
	Statistics	χ^2=5.627; p=0.060		
Correctional facility	Yes	136 (54.0%)	111 (60.3%)	42 (58.3%)
	No	116 (46.0%)	73 (39.7%)	30 (41.7%)

	Statistics	χ^2=1.824; p=0.402		
Delinquency adjudications	Yes	68 (50.0%)	53 (47.7%)	17 (40.5%)
	No	68 (50.0%)	58 (52.3%)	25 (59.5%)
	Statistics	χ^2=1.167; p=0.558		
Smoking	Yes	108 (79.4%)	90 (81.1%)	35 (83.3%)
	No	28 (20.6%)	21 (18.9%)	7 (16.7%)
	Statistics	χ^2=0.340; p=0.844		

The data are represented as numbers and frequencies. Haplotypes - *COMT* rs4818-rs4680 CA, GG and CG haplotypes

Supplementary Table S4. The PCL-YV total scores as well as the scores on interpersonal (F1), affective (F2), behavioral (F3) and criminal (F4) domains in participants with CD and control subjects from the correctional facility, carrying *COMT* rs4818-rs4680 CA, GG and CG haplotypes.

PCL-YV scores	Group	***COMT* rs4818-rs4680 haplotypes**			Statistics
		**CA**	**GG**	**CG**	
Total	CD	27 (18;32)	25 (18;31)	21 (18;29)	H=1.04; p=0.596
	Control	11 (7;15)	12 (10;15)	10 (8;16)	H=0.53; p=0.768
F1 domain	CD	5 (4;7)	5 (4;8)	4 (3;6)	H=3.05; p=0.218
	Control	1 (1;3)	2 (1;3)	2 (0;3)	H=1.34; p=0.512
F2 domain	CD	7 (5;9)	7 (5;10)	6 (4;8)	H=1.60; p=0.450
	Control	4 (2;5)	3 (2;4)	3 (1;5)	H=0.36; p=0.837
F3 domain	CD	6 (4;8)	6 (4;7)	6 (4;7)	H=0.98; p=0.612
	Control	2 (0;4)	2 (2;4)	4 (2;6)	H=1.65; p=0.438
F4 domain	CD	7 (5;8)	7 (5;8)	7 (5;8)	H=0.47; p=0.789
	Control	3 (2;4)	3 (2;5)	4 (2;4)	H=1.60; p=0.449

The data are represented as medians and interquartile ranges, while significant p values are denoted in bold. CD – conduct disorder; haplotypes - *COMT* rs4818-rs4680 CA, GG and CG haplotypes; F1 domain - interpersonal domain scores of the PCL-YV; F2 domain – affective domain scores of the PCL-YV; F3 domain – behavioral domain scores of the PCL-YV; F4 domain – criminal domain scores of the PCL-YV; PCL-YV - The Hare Psychopathy Checklist - Youth Version.

Supplementary Table S5. The MOAS total scores and scores on total aggression, verbal aggression, physical aggression towards objects and physical aggression towards others, auto-aggression, suicidality, irritability, subjective and open irritability domains in participants with CD and control subjects from the correctional facility, carrying various *COMT* rs4818-rs4680 CA, GG and CG haplotypes.

MOAS scores	Group	***COMT* rs4818-rs4680 haplotypes**			Statistics
		**CA**	**GG**	**CG**	
Total	CD	32 (20;42)	29 (17;37)	34 (17;47)	H=2.74; p=0.255
	Control	13 (4;19)	18 (10;23)	18 (6;25)	H=2.27; p=0.322
Aggression (total) scores	CD	24 (15;35)	21 (11;30)	28 (12;39)	H=2.37; p=0.306
	Control	8 (1;12)	10 (6;12)	12 (0;20)	H=1.46; p=0.482
Verbal aggression scores	CD	8 (6;10)	7 (4;10)	10 (4;10)	H=4.69; p=0.096
	Control	4 (1;6)	6 (1;7)	6 (0;7)	H=1.92; p=0.382
Physical aggression scores toward objects	CD	6 (2;10)	2 (2;8)	4 (2;6)	H=1.87; p=0.393
	Control	2 (0;3)	2 (0;2)	2 (0;2)	H=1.51; p=0.469
Auto-aggression scores	CD	0 (0;6)	0 (0;6)	6 (0;9)	H=1.81; p=0.404
	Control	0 (0;0)	0 (0;0)	0 (0;6)	H=1.86; p=0.395
	CD	9 (3;9)	6 (3;9)	9 (3;9)	H=0.67; p=0.715

Physical aggression scores towards others	Control	2 (0;3)	3 (2;3)	3 (0;6)	H=3.12; p=0.210
Irritability (total) scores	CD	6 (5;8)	6 (5;7)	6 (5;7)	H=4.88; p=0.087
	Control	4 (2;6)	5 (3;6)	4 (3;6)	H=1.35; p=0.509
Subjective irritability scores	CD	3 (3;4)	3 (2;4)	3 (3;3)	H=3.05; p=0.217
	Control	2 (1;3)	3 (2;3)	3 (2;3)	H=1.84; p=0.400
Open irritability scores	CD	3 (2;4)	3 (2;4)	3 (2;4)	H=4.67; p=0.097
	Control	1 (1;3)	2 (1;3)	1 (1;3)	H=1.14; p=0.567
Suicidality (total) scores	CD	1 (0;2)	1 (0;1)	1 (0;1)	H=1.48; p=0.477
	Control	1 (0;1)	0 (0;1)	1 (0;1)	H=0.14; p=0.934

The data are represented as median and interquartile range, while significant p values are denoted in bold. CD – conduct disorder; haplotypes - *COMT* rs4818-rs4680 CA, GG and CG haplotypes; MOAS – The Modified Overt Aggression Scale.
